# Supplementary material for: Association between alanine aminotransferase within the normal range and all-cause and cause-specific mortality: A nationwide cohort study
Source: PLoS One. 2020 Nov 20;15(11):e0242431. doi: 10.1371/journal.pone.0242431 (PMC7678955; doi:10.1371/journal.pone.0242431)
Supplement: S1 Table — (DOCX) [file pone.0242431.s001.docx]

| S1 Table – Baseline Characteristics by ALT Quartile in Females | | | | | | | | | |
| --- | --- | --- | --- | --- | --- | --- | --- | --- | --- |
|  | Overall | Quartile 1 | Quartile 2 | Quartile 3 | Quartile 4 | P-value^*^  (1 vs. 2) | P-value^*^  (1 vs. 3) | P-value^*^  (1 vs. 4) | Overall^#^  P-value |
| Demographics |  |  |  |  |  |  |  |  |  |
| Age, Mean (SD) | 41.7 (0.52) | 40.6 (0.80) | 41.6 (0.83) | 41.7 (0.67) | 43.9 (0.56) | 0.321 | 0.196 | 0.0006 | 0.0003 |
| Race/Ethnicity |  |  |  |  |  |  |  |  |  |
| White | 1391 (78%) | 279 (74%) | 350 (79%) | 391 (81%) | 371 (75%) |  |  |  | <0.0001 |
| Black | 1045 (11%) | 338 (18%) | 248 (10%) | 215 (8%) | 244 (10%) |  |  |  |  |
| Mexican American | 885 (4%) | 143 (3%) | 181 (4%) | 232 (4%) | 329 (6%) |  |  |  |  |
| Other | 140 (7%) | 20 (5%) | 38 (7%) | 38 (7%) | 44 (9%) |  |  |  |  |
| Poverty-Income Ratio | 3.07 (0.08) | 2.73 (0.12) | 3.08 (0.11) | 3.10 (0.12) | 3.31 (0.12) | 0.0076 | 0.012 | 0.0001 | 0.0009 |
| Sociobehavioral Factors |  |  |  |  |  |  |  |  |  |
| Smoking Status | 1320 (45%) | 301 (49%) | 330 (47%) | 336 (46%) | 353 (39%) |  |  |  | 0.0147 |
| Alcohol consumption | 1.22 (0.12) | 1.3 (0.3) | 0.9 (0.2) | 1.7 (0.2) | 0.9 (0.1) | 0.17 | 0.17 | 0.25 | 0.015 |
| Exercise - jog/run in the past month? | 286 (8.7%) | 69 (9%) | 67 (9.7%) | 70 (7.1%) | 80 (9.2%) |  |  |  | 0.6389 |
| Carbohydrate Intake (kcal) | 914 (12) | 931 (19) | 890 (23) | 909 (19) | 917 (22) | 0.1759 | 0.3975 | 0.6678 | 0.5611 |
| Protein Intake (kcal) | 268 (3.8) | 259 (5.5) | 264 (6.6) | 261 (5.6) | 273 (7.4) | 0.5046 | 0.7966 | 0.1487 | 0.4559 |
| Total fat intake (kcal) | 621 (9.6) | 629 (18) | 626 (17) | 619 (16) | 603 (19) | 0.9092 | 0.6776 | 0.3517 | 0.775 |
| Total caloric intake (kcal) | 1780 (21) | 1799 (36) | 1757 (40) | 1768 (34) | 1768 (40) | 0.4236 | 0.518 | 0.5885 | 0.8671 |
| Examination Measurements |  |  |  |  |  |  |  |  |  |
| Heart Rate (beats-per-min) | 82.6 (2.2) | 80.8 (3.6) | 87.2 (6.4) | 80.6 (4.3) | 82.8 (3.8) | 0.3893 | 0.9811 | 0.6873 | 0.7738 |
| BMI (kg/m^2^) | 25.6 (0.154) | 24.6 (0.213) | 24.9 (0.261) | 25.7 (0.304) | 27.0 (0.333) | 0.2448 | 0.0099 | <0.0001 | <0.0001 |
| Waist Circumference (cm) | 86.0 (0.38) | 83.4 (0.526) | 84.1 (0.668) | 86.4 (0.742) | 89.2 (0.745) | 0.2849 | 0.0028 | <0.0001 | <0.0001 |
| Thigh Circumference (cm) | 50.9 (0.155) | 50.2 (0.251) | 50.6 (0.267) | 51.1 (0.354) | 51.9 (0.399) | 0.2756 | 0.0882 | 0.001 | 0.0101 |
| Systolic Blood Pressure (mmHg) | 116.5 (0.6) | 114.7 (0.9) | 115.9 (0.8) | 117 (0.7) | 118.4 (0.8) | 0.2308 | 0.0315 | 0.0017 | 0.0114 |
| Diastolic Blood Pressure (mmHg) | 71.3 (0.3) | 70.5 (0.40) | 70.7 (0.4) | 71.7 (0.4) | 72.6 (0.4) | 0.7048 | 0.0389 | 0.0002 | 0.0002 |
| Laboratory Measurements |  |  |  |  |  |  |  |  |  |
| Plasma Glucose (mg/dl) | 93.0 (0.6) | 92.5 (1.4) | 91.5 (0.9) | 93.2 (1.1) | 95.1 (1.0) | 0.5917 | 0.7366 | 0.1476 | 0.0356 |
| Hemoglobin A1c (%) | 5.19 (0.02) | 5.19 (0.04) | 5.17 (0.03) | 5.16 (0.04) | 5.27 (0.04) | 0.6044 | 0.5853 | 0.1364 | 0.0237 |
| Serum Insulin (uU/mL) | 9.0 (0.2) | 7.8 (0.2) | 8.5 (0.4) | 9.2 (0.4) | 10.4 (0.4) | 0.12 | 0.0039 | <0.0001 | <0.0001 |
| Serum HDL (mg/dl) | 55.9 (0.5) | 54.9 (0.7) | 56.6 (0.9) | 55.5 (0.9) | 55.6 (0.7) | 0.1102 | 0.5824 | 0.5282 | 0.3752 |
| Serum LDL (mg/dl) | 124 (1.5) | 120 (2.4) | 121 (2.6) | 125 (2.3) | 128 (2.3) | 0.7722 | 0.0945 | 0.011 | 0.0213 |
| Serum triglycerides (mg/dl) | 115 (1.8) | 103 (2.9) | 106 (3.9) | 119 (4.0) | 126 (3.4) | 0.5726 | 0.0019 | <0.0001 | <0.0001 |
| ALT (U/L) | 11.3 (0.2) | 6.7 (0.1) | 9.5 (0.02) | 12.0 (0.05) | 16.2 (0.10) | <0.0001 | <0.0001 | <0.0001 | <0.0001 |
| AST (U/L) | 17.1 (0.1) | 14.7 (0.2) | 16.1 (0.2) | 17.6 (0.2) | 19.6 (0.2) | <0.0001 | <0.0001 | <0.0001 | <0.0001 |
| GGT (U/L) | 18.0 (0.3) | 14.3 (0.3) | 15.5 (0.3) | 18.0 (0.4) | 23.6 (0.6) | 0.0089 | <0.0001 | <0.0001 | <0.0001 |
| LDH (U/L) | 151 (1.9) | 139.6 (2.4) | 147.2 (2.3) | 151.5 (2.2) | 163.0 (2.2) | 0.0095 | <0.0001 | <0.0001 | <0.0001 |
| Serum Albumin (g/dL) | 4.09 (0.02) | 4.06 (0.03) | 4.11 (0.03) | 4.11 (0.03) | 4.10 (0.02) | 0.1493 | 0.1911 | 0.3527 | 0.496 |
| Total Bilirubin | 0.512 (0.01) | 0.476 (0.01) | 0.507 (0.01) | 0.536 (0.02) | 0.536 (0.01) | 0.0777 | 0.0031 | 0.0074 | 0.0174 |
| Serum ferritin (ng/mL) | 63.7 (1.7) | 47.2 (2.7) | 55.6 (2.6) | 66.9 (3.2) | 80.9 (5.4) | 0.029 | <0.0001 | <0.0001 | <0.0001 |
| Serum C-reactive protein (mg/dL) | 0.411 (0.02) | 0.387 (0.02) | 0.405 (0.04) | 0.40 (0.03) | 0.443 (0.02) | 0.6496 | 0.7039 | 0.1046 | 0.4132 |
| Serum Creatinine | 0.956 (0.01) | 0.959 (0.01) | 0.964 (0.01) | 0.949 (0.01) | 0.954 (0.01) | 0.6431 | 0.4161 | 0.6325 | 0.4964 |
| Hemoglobin | 13.2 (0.03) | 13.0 (0.05) | 13.2 (0.05) | 13.3 (0.06) | 13.4 (0.06) | 0.0012 | 0.0002 | <0.0001 | <0.0001 |
| Platelets | 279 (3) | 278 (3) | 282 (4) | 276 (4) | 277 (4) | 0.3112 | 0.7164 | 0.7788 | 0.5706 |
| TSH | 2.17 (0.11) | 2.25 (0.25) | 2.40 (0.34) | 1.99 (0.09) | 2.19 (0.15) | 0.7367 | 0.2964 | 0.8542 | 0.2957 |
| Comorbidities |  |  |  |  |  |  |  |  |  |
| Diabetes (%) | 292 (4.6%) | 53 (3.5%) | 52 (3.4%) | 75 (4.5%) | 112 (6.8%) |  |  |  | 0.045 |
| Impaired Glucose Tolerance (%) | 1130 (23%) | 230 (19%) | 242 (20%) | 278 (20%) | 380 (30%) |  |  |  | <0.0001 |
| Hypertension (%) | 802 (19%) | 164 (17%) | 176 (15%) | 216 (19%) | 246 (23%) |  |  |  | 0.0173 |
| Metabolic Syndrome (%) | 843 (20%) | 149 (15%) | 159 (14%) | 231 (24%) | 304 (25%) |  |  |  | <0.0001 |
| HOMA-IR (%) | 908 (15%) | 149 (9.9%) | 171 (12%) | 229 (15%) | 359 (23%) |  |  |  | <0.0001 |
| Albuminuria (%) | 518 (12%) | 128 (13%) | 111 (11%) | 131 (11%) | 148 (11%) |  |  |  | 0.8244 |
| History of MI | 57 (1.2%) | 12 (2%) | 19 (1.3%) | 12 (0.7%) | 14 (1.1%) |  |  |  | 0.0617 |
| History of Stroke | 42 (1%) | 8 (1.6%) | 15 (1.2%) | 8 (0.3%) | 11 (1%) |  |  |  | 0.13 |
| Continuous variables presented as mean (standard deviation); categorical variables presented as Number (weighted %). Abbreviations: HOMA-IR = Homeostatic Model Assessment of Insulin Resistance, MI = Myocardial Infarction, CHF = Congestive Heart Failure.  ^#^Overall p-value calculated using Rao-Scott design-adjusted Chi-square test for categorical variables and one-way ANOVA for continuous variables.  ^*^For continuous variables, post-hoc pairwise Tukey comparison tests were conducted. Adjustment for multiple comparisons was not done as comparisons were explorative and did not guide our choice of covariates to include in the analysis. | | | | | | | | | |
