# Supplementary material for: Association between alanine aminotransferase within the normal range and all-cause and cause-specific mortality: A nationwide cohort study
Source: PLoS One. 2020 Nov 20;15(11):e0242431. doi: 10.1371/journal.pone.0242431 (PMC7678955; doi:10.1371/journal.pone.0242431)
Supplement: S2 Table — (DOCX) [file pone.0242431.s002.docx]

| S2 Table: Association between ALT as a Continuous Variable and All-cause and Cause-specific Mortality in Males | | | | | | | |
| --- | --- | --- | --- | --- | --- | --- | --- |
|  |  |  | Model 1 | | Model 2 | Model 3 | Model 4 |
|  |  | | HR (95% CI) | | HR (95% CI) | HR (95% CI) | HR (95% CI) |
| All-Cause Mortality | ALT | **Deaths (%)^#^:**  940 (33%) | 0.91 (0.87, 0.96) | | 0.98 (0.96, 1.00) | 0.97 (0.95, 0.99) | 0.97 (0.95, 0.99) |
|  |  | |  | |  |  |  |
| 10-Year All-Cause Mortality | ALT | **Deaths (%):**  309 (11%) | 0.91 (0.87, 0.96) | | 0.96 (0.92, 1.01) | 0.97 (0.94, 1.01) | 0.96 (0.93, 0.997) |
|  |  | | |  |  |  |  |
| Cardiovascular Mortality | ALT | **Deaths (%):**  249 (6.3%) | 0.97 (0.93, 1.00) | | 1.02 (0.98, 1.05) | 1.01 (0.98, 1.05) | 0.99 (0.96, 1.04) |
|  |  | |  | |  |  |  |
| Cancer-related Mortality | ALT | **Deaths (%):**  214 (6.1%) | 0.91 (0.87, 0.94) | | 0.94 (0.90, 0.98) | 0.95 (0.91, 0.99) | 0.98 (0.93, 1.03) |
| Abbreviations: HR = Hazard Ratio, CI = Confidence Interval.  ^#^Deaths represented as number of deaths (weighted % of sample)  Model 1 = unadjusted model  Model 2 adjusted for demographic (age, poverty-income ratio, race/ethnicity) and sociobehavioral covariates (alcohol, smoking status)  Model 3 adjusted for Model 2 covariates + cardiometabolic covariates (waist circumference, HDL, systolic BP, triglycerides, C-reactive protein, albuminuria, history of CVD condition)  Model 4 adjusted for Model 3 covariates + liver function-related covariates (albumin, platelet count, AST, total bilirubin) | | | | | | | |
