# Supplementary material for: Association between alanine aminotransferase within the normal range and all-cause and cause-specific mortality: A nationwide cohort study
Source: PLoS One. 2020 Nov 20;15(11):e0242431. doi: 10.1371/journal.pone.0242431 (PMC7678955; doi:10.1371/journal.pone.0242431)
Supplement: S3 Table — (DOCX) [file pone.0242431.s003.docx]

| S3 Table: Association between ALT as a Continuous Variable and All-cause and Cause-specific Mortality in Females | | | | | | | |
| --- | --- | --- | --- | --- | --- | --- | --- |
|  |  |  | Model 1 | | Model 2 | Model 3 | Model 4 |
|  |  | | HR (95% CI) | | HR (95% CI) | HR (95% CI) | HR (95% CI) |
| All-Cause Mortality | ALT | **Deaths (%)^#^:**  861 (23%) | 0.99 (0.97, 1.03) | | 0.97 (0.95, 1.00) | 0.96 (0.93, 0.98) | 0.94 (0.91, 0.97) |
|  |  | |  | |  |  |  |
| 10-Year All-Cause Mortality | ALT | **Deaths (%):**  243 (6.5%) | 0.95 (0.91, 1.00) | | 0.94 (0.90, 0.99) | 0.93 (0.88, 0.98) | 0.90 (0.85, 0.95) |
|  |  | | |  |  |  |  |
| Cardiovascular Mortality | ALT | **Deaths (%):**  234 (6.2%) | 0.99 (0.94, 1.06) | | 0.97 (0.92, 1.03) | 0.94 (0.89, 0.99) | 0.95 (0.86, 1.00) |
|  |  | |  | |  |  |  |
| Cancer-related Mortality | ALT | **Deaths (%):**  220 (5.8%) | 0.96 (0.91, 1.01) | | 0.94 (0.89, 0.99) | 0.94 (0.89, 0.99) | 0.95 (0.88, 1.01) |
| Abbreviations: HR = Hazard Ratio, CI = Confidence Interval.  ^#^Deaths represented as number of deaths (weighted % of sample)  Model 1 = unadjusted model  Model 2 adjusted for demographic (age, poverty-income ratio, race/ethnicity) and sociobehavioral covariates (alcohol, smoking status)  Model 3 adjusted for Model 2 covariates + cardiometabolic covariates (waist circumference, HDL, systolic BP, triglycerides, C-reactive protein, albuminuria, history of CVD condition)  Model 4 adjusted for Model 3 covariates + liver function-related covariates (albumin, platelet count, AST, total bilirubin) | | | | | | | |
