# Supplementary material for: Association between alanine aminotransferase within the normal range and all-cause and cause-specific mortality: A nationwide cohort study
Source: PLoS One. 2020 Nov 20;15(11):e0242431. doi: 10.1371/journal.pone.0242431 (PMC7678955; doi:10.1371/journal.pone.0242431)
Supplement: Supplementary file 5 [file pone.0242431.s005.docx]

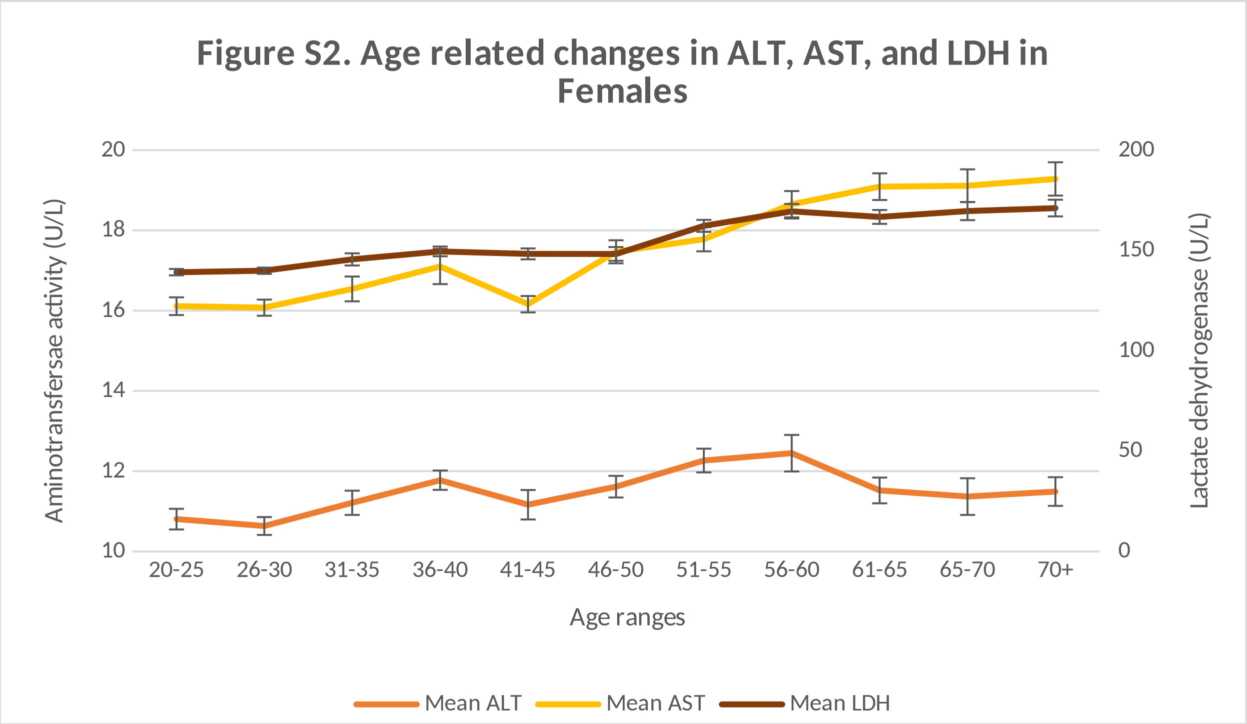
Figure S2. Age related changes in ALT, AST, and LDH in Females
Aminotransfersae activity (U/L)
20
18
16
14
12
10
200
150
100
50
0
Lactate dehydrogenase (U/L)
20-25
26-30
31-35
36-40
41-45
46-50
51-55
56-60
61-65
65-70
70+
Age ranges
Mean ALT
Mean AST
Mean LDH
